# Mycotic Aneurysm in Salmonella enteritidis Infection: A Fatal Complication

**DOI:** 10.7759/cureus.75739

**Published:** 2024-12-15

**Authors:** Grace Gui Jin Khoo, Paul Andrew Chong Yew Ong, Joel Kae Wei Wong, Muhamad Aibaq B Muhamad Yazid, Chee Yik Chang

**Affiliations:** 1 Internal Medicine, Hospital Sultanah Aminah, Johor Bahru, MYS; 2 Radiology, Hospital Sultanah Aminah, Johor Bahru, MYS; 3 Infectious Diseases, Hospital Sultanah Aminah, Johor Bahru, MYS

**Keywords:** mycotic aneurysms, nontyphoidal salmonella, salmonella aortitis, salmonella enteritidis, septic shock (ss)

## Abstract

Mycotic aneurysms are rare but severe complications that can arise from systemic bacterial infections, including those caused by Salmonella species. These aneurysms can progress rapidly and are associated with high mortality. A 62-year-old man with poorly controlled type 2 diabetes mellitus presented to the hospital in septic shock. The blood culture grew Salmonella enteritidis. A computed tomography angiogram showed a ruptured mycotic aneurysm of the infrarenal abdominal aorta. Despite antibiotic therapy and supportive care, the patient's condition rapidly deteriorated, resulting in his death, most likely due to aneurysm rupture. This case highlights the importance of vigilance in patients with Salmonella bacteremia, particularly in high-risk individuals, such as the elderly and people with underlying cardiovascular diseases. Early imaging and timely intervention are critical for improving outcomes and preventing fatal complications, such as aneurysm rupture.

## Introduction

Salmonella species are a leading cause of foodborne illness worldwide, responsible for both typhoidal and non-typhoidal infections. While most non-typhoidal Salmonella (NTS) infections result in self-limited gastroenteritis in immunocompetent individuals, they can lead to severe systemic infections in immunocompromised patients, such as those with diabetes, cancer, or HIV [1,2]. In these populations, invasive Salmonella infections, such as bacteremia and endovascular involvement, pose significant risks and may lead to complications including endocarditis, osteomyelitis, and mycotic aneurysms [3].

Mycotic aneurysms, which occur due to infection of the arterial wall, are a rare but potentially fatal complication of Salmonella bacteremia. These aneurysms develop in the presence of pre-existing vascular lesions, such as atherosclerotic plaques, which predispose patients to bacterial colonization and subsequent infection. Although most mycotic aneurysms are found in the abdominal aorta, other vessels may also be affected [4]. Patients with risk factors such as advanced age, poorly controlled diabetes, hypertension, and atherosclerosis are at increased risk of developing mycotic aneurysms in the setting of Salmonella bacteremia [5].

Diabetes mellitus is a well-known risk factor for severe Salmonella infections because it impairs the immune response in these patients. Poorly controlled diabetes mellitus can lead to frequent bacterial infections, and in the presence of Salmonella bacteremia, the risk of vascular infections is significantly increased [2]. Prompt recognition, diagnosis, and treatment of mycotic aneurysms are critical to preventing rupture and death, but the condition is often difficult to manage, particularly in patients with multiple comorbidities [6]. This report describes a 62-year-old man with uncontrolled diabetes mellitus who succumbed due to a ruptured abdominal aortic mycotic aneurysm caused by invasive Salmonella enteritidis infection.

## Case presentation

A 62-year-old man with a long-standing history of poorly controlled type 2 diabetes mellitus presented to the emergency department with a one-week history of lethargy, vomiting, and loss of appetite. On examination, he appeared lethargic but alert and conscious, with a Glasgow Coma Scale score of 15/15. He was afebrile but hypotensive, with a blood pressure of 80/47 mmHg and a pulse rate of 101 beats per minute. Abdominal examination revealed tenderness in the right lumbar region, although renal punch was negative. Cardiovascular and respiratory findings were otherwise unremarkable.

Initial laboratory investigations revealed significant hyperglycemia (random blood sugar: 34.5 mmol/L; reference range: 3.6-8 mmol/L), normochromic normocytic anemia (hemoglobin: 11.7 g/dL; reference range: 13-17 g/dL), leukocytosis (white blood cell count: 16.2 x 10^9/L; reference range: 4-10 x 10^9/L), thrombocytopenia (platelet count: 13 x 10^9/L; reference range: 150-410 x 10^9/L), elevated C-reactive protein (165.6 mg/L; reference range: <5 mg/L), and renal impairment (urea: 41.3 mmol/L; reference range: 3-9.2 mmol/L, creatinine: 204 µmol/L; reference range: 64-104 µmol/L). Based on these findings, a clinical diagnosis of septic shock, likely secondary to a urinary tract infection, was made. The patient was initiated on intravenous fluids and intravenous amoxicillin-clavulanic acid 1.2 g daily. However, despite aggressive fluid resuscitation, he remained hypotensive and required a noradrenaline infusion at 0.1 mcg/kg/min to maintain blood pressure.

Abdominal ultrasonography performed on day two of admission revealed bilateral renal parenchymal disease with minimal free fluid in the perihepatic region. On day three, blood cultures grew Salmonella spp., later identified as Salmonella Enteritidis. The isolate was sensitive to ampicillin, ceftriaxone, bactrim, and ciprofloxacin. Hence, the antibiotic therapy was changed to intravenous ceftriaxone 2 g daily. Despite this, on day four, the patient’s condition further deteriorated; his hemoglobin dropped significantly from 11.7 g/dL to 8.5 g/dL, with no overt signs of external bleeding. A detailed physical examination revealed a pulsatile mass in the epigastric region, although no bruit was heard upon auscultation.

**Figure 1:**
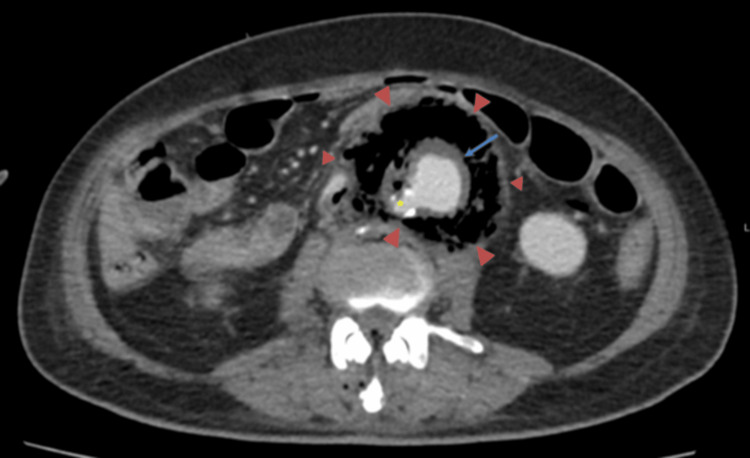
An axial view of the CTA aorta showing a periaortic collection that is filled with air locules (red arrowheads). The abdominal aorta with atherosclerosis (yellow asterisk) with a saccular aneurysmal dilatation (blue arrow) is noted. CTA: Computed tomography angiography

**Figure 2:**
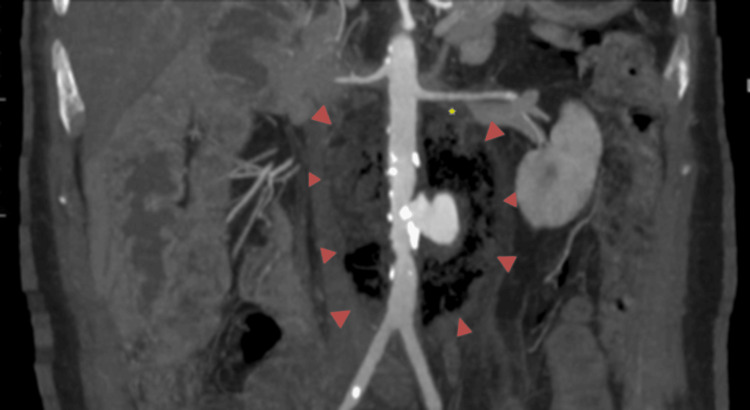
A maximum intensity projection image in the coronal view of the CTA aorta showing the infrarenal periaortic collection (red arrowheads) with mycotic aneurysm, in relation to the renal vessels and aortic bifurcation. No clear plane between this collection with the left renal vein. Thrombus seen within this vessel (yellow asterisk). CTA: Computed tomography angiography

A diagnosis of a mycotic aneurysm secondary to Salmonella infection was suspected. An urgent computed tomography angiogram revealed a saccular infrarenal abdominal aortic aneurysm measuring 2.1 cm in diameter, accompanied by surrounding air locules and a collection suggestive of an infected (mycotic) aneurysm. The aneurysm was enveloped by a collection of air locules, measuring approximately 5.8 x 7.2 x 9.5 cm. Additionally, filling defects with air locules were observed in the inferior vena cava, left renal vein, and right common iliac vein, indicating the potential spread of infection (Figures 1, 2).

The patient was referred for surgical evaluation, but after a thorough assessment, he was deemed unsuitable for surgical intervention due to his unstable condition and the significant risks associated with the procedure. Despite the medical team's best efforts to stabilize him, his condition continued to deteriorate. Later that day, he succumbed, most likely due to the rupture of the abdominal aortic aneurysm.

## Discussion

Salmonellae are classified into typhoidal (S. enterica serotypes Typhi and Paratyphi A) and nontyphoidal (NTS) serotypes. The two most common NTS serotypes isolated from humans were S. enterica ser. Typhimurium and S. enterica ser. Enteritidis. Non-typhoidal Salmonella infections can cause a variety of clinical manifestations, including gastroenteritis, bacteremia, septic arthritis, osteomyelitis, endovascular infection, and even intestinal perforation [7].

Invasive Salmonella infections are more common in the elderly and people with underlying cardiovascular diseases such as atherosclerosis, hypertension, and diabetes. These conditions promote bacterial seeding in vascular lesions, which leads to vascular complications like mycotic aneurysms [4]. In this case, poorly controlled diabetes and advanced age are significant risk factors for the development of a mycotic aneurysm due to invasive Salmonella infection.

Salmonella bacteremia can present with fever and gastrointestinal symptoms, as well as signs of septic shock-like hypotension and tachycardia, especially in immunocompromised patients [3]. The patient presented with sepsis symptoms, such as lethargy, vomiting, and hypotension. Blood cultures confirmed the diagnosis of Salmonella Enteritidis bacteremia, prompting the initiation of antimicrobial treatment. However, bacteremia in the context of Salmonella infections, particularly in high-risk individuals, increases the likelihood of secondary complications such as vascular infections.

Mycotic aneurysms should be suspected in patients with Salmonella bacteremia who develop abdominal pain, hypotension, and a pulsatile mass. In this case, the patient had a sudden drop in hemoglobin and was found to have a pulsatile abdominal mass, raising suspicion of a vascular complication. Imaging studies, such as computed tomography angiography, are crucial to confirm the presence of mycotic aneurysms and determine the extent of vascular involvement [5].

Mycotic aneurysms are a rare but severe complication of Salmonella bacteremia. They are caused by bacterial seeding of the arterial wall, which often occurs in the presence of pre-existing atherosclerosis or other vascular lesions. Once bacteria colonize the vessel wall, they cause an inflammatory response that weakens the arterial structure, resulting in aneurysm formation [6]. The abdominal aorta is the most common location for these aneurysms, but they can also occur in other vessels. Advanced age and cardiovascular risk factors, such as hypertension and atherosclerosis, are common risk factors for the development of mycotic aneurysms, as was the case for this patient.

Salmonella infections are known to have a predilection for the vascular system in people who have atherosclerosis. Guo et al. emphasized the importance of considering this diagnosis in patients with bacteremia and risk factors for vascular disease in their review of mycotic aneurysms caused by Salmonella species [5]. The mortality rates associated with non-typhoidal Salmonella-caused mycotic aneurysms are high when diagnosis or treatment is delayed [4].

Mycotic aneurysms are treated with a combination of antibiotics and surgery in appropriate cases. Antibiotics should be selected based on culture sensitivities, and treatment should last at least six weeks to ensure infection eradication. Following the identification of Salmonella Enteritidis in blood cultures, the patient was given ceftriaxone, as is recommended for invasive Salmonella infections [3,8].

Surgical intervention such as resection of the infected aneurysm and vascular reconstruction is frequently required to prevent rupture. Endovascular aneurysm repair (EVAR) has recently emerged as a less invasive alternative to open surgery for the treatment of both non-infected and infected aneurysms. EVAR involves inserting a catheter through the femoral artery to place a stent graft inside the aneurysm. This procedure keeps the aneurysm out of circulation, preventing rupture without the need for extensive surgical dissection [4]. EVAR has shown promising results in the treatment of mycotic aneurysms, particularly in high-risk patients who are unsuitable for open surgery due to advanced age, frailty, or multiple comorbidities [9].

Unfortunately, in this case, the patient's rapidly deteriorating condition made surgical intervention impossible. Multiple studies have shown that the mortality rate for untreated or ruptured mycotic aneurysms is extremely high, and prompt diagnosis is critical to improving outcomes [5,6]. Delayed diagnosis and intervention in mycotic aneurysms, particularly those caused by Salmonella, are associated with a poor prognosis and a high mortality rate [9].

## Conclusions

This case demonstrates the critical importance of maintaining a high level of clinical suspicion for mycotic aneurysm in high-risk patients with non-typhoidal Salmonella bacteremia. To avoid fatal outcomes, timely diagnosis using blood cultures and imaging, as well as appropriate antimicrobial and surgical interventions, is critical.
